# Risk Factors for Adverse Pregnancy Outcomes in Reduced Fetal Movement: An IPD Meta‐Analysis

**DOI:** 10.1111/1471-0528.18132

**Published:** 2025-03-17

**Authors:** Yongyi Lu, Victoria Palin, Alexander Heazell

**Affiliations:** ^1^ Maternal and Fetal Health Research Centre, Division of Developmental Biology and Medicine, Faculty of Biology, Medicine and Health The University of Manchester Manchester UK; ^2^ Saint Mary's Hospital Manchester University NHS Foundation Trust Manchester UK

**Keywords:** adverse pregnancy outcome, complications, decreased fetal movement, fetal growth restriction, fetal surveillance, IPD‐MA, perinatal mortality, placenta, stillbirth

## Abstract

**Objective:**

Women experiencing reduced fetal movements (RFM) have an increased risk of adverse pregnancy outcomes (APO). This study aimed to identify factors most associated with APO in RFM pregnancies.

**Design:**

Individual participant data meta‐analysis (IPD‐MA).

**Setting:**

Multiple maternity units across the UK.

**Population or Sample:**

1175 singleton pregnancies with RFM between 28^+0^ and 41^+0^ weeks' gestation from four prospective cohorts and two randomised controlled trials (RCTs).

**Methods:**

Factors associated with APO were assessed using two‐stage IPD‐MA.

**Main Outcome Measures:**

A composite adverse pregnancy outcome, including: adjusted Odds Ratio, stillbirth, fetal growth restriction (FGR, birthweight ≤ 3rd centile) and neonatal intensive care unit (NICU) admission.

**Main Results:**

APO occurred in 7.7% of RFM pregnancies, with FGR being the most common complication (4.6%). The strongest associations with APO were observed for abnormal fetal heart rate (adjusted Odds Ratio (aOR) = 3.65, 95% CI: 1.84–7.23), cigarette smoking (aOR = 2.96, 95% CI: 1.36–6.44) and maternal past medical history (aOR = 2.35, 95% CI: 1.14–4.82). Lower estimated fetal weight (EFW) centile was also significantly associated with APO (aOR = 0.97, 95% CI: 0.95–0.99), though substantial heterogeneity was present between studies (*I* ^2^ = 80.74%, *Q*‐statistic: *p* < 0.001).

**Conclusions:**

IPD‐MA enabled the synthesis of individual‐level data across studies, allowing for more accurate and reliable associations by accounting for heterogeneity. Further work is required to investigate the model's generalisability across diverse populations.

**Abbreviations**
AFI: amniotic fluid index
AFM: absent fetal movements
APO: adverse pregnancy outcome
CTG: cardiotocography
DBP: diastolic blood pressure
EFW: estimated fetal weight
FGR: fetal growth restriction
IPD‐MA: individual participant data meta‐analysis
MAR: missing at random
MCAR: missing completely at random
MI: multiple imputation
NICU: neonatal intensive care unit
RCT: randomised controlled trial
RFM: reduced fetal movement
SBP: systolic blood pressure

## 1 Introduction

Pregnancies in which there is subjective perception of reduced fetal movements (RFM) are more likely to end in adverse pregnancy outcomes, including stillbirth (approximately 2.3‐fold increased risk) [1] and fetal growth restriction (FGR) which occurs in 20%–23% of cases [2, 3, 4, 5]. Up to half of stillbirths report RFM before the diagnosis of intrauterine fetal death [6, 7, 8, 9]. An individual participant data (IPD) meta‐analysis of case–control studies confirmed that RFM is a significant risk factor for stillbirth in both late preterm and term pregnancies but suggested the greatest risk was from 28 to 32 weeks' or in babies that were small for gestational age [1, 10, 11]. In addition, interventions following RFM can lead to increased rates of induction of labour (IOL), caesarean section and neonatal unit admission [12, 13, 14, 15]. Some of this intervention is likely to result from actions taken by clinicians in response to RFM, aimed at preventing rare, serious outcomes such as stillbirth and perinatal asphyxia. Thus, identifying factors that are most associated with adverse pregnancy outcomes would help clinicians identify women at greater risk who present with RFM, enabling them to provide personalised appropriate interventions.

Education on RFM may encourage women to present earlier in gestation for improved monitoring [16]; aggregate data meta‐analysis suggests this approach may be associated with a reduction in neonatal intensive care unit admissions and Apgar scores of < 7 at 5 min of age [17]. Furthermore, individual studies found factors related to fetal growth and placental health are closely linked to poor pregnancy outcomes following maternal perception of RFM [2, 5, 15]. However, these findings drawn from single‐population studies might be susceptible to centre‐specific biases, affected by patient populations, smaller sample sizes and unit‐specific guidelines. This imbalance can influence the robustness of the comparison and may not provide reliable effect estimates. Therefore, the impact of the factors on adverse pregnancy outcomes is still uncertain. This research primarily aimed to identify the most predictive factors of adverse pregnancy outcomes in women presenting with RFM using individual patient data meta‐analysis.

## 2 Methods

This Individual Patient Data Meta‐Analysis (IPD‐MA) adhered to the applicable section of the Preferred Reporting Items for Systematic Reviews and Meta‐Analysis of Individual Patients Data (PRISMA‐IPD) guidelines [18].

### 2.1 Identification of Studies and Eligibility Criteria

A literature search in Medline and EMBASE Database of clinical trials was conducted to identify eligible individual studies of pregnant women with RFM. Titles and abstracts were screened, and the full text of the potentially eligible articles was reviewed against the following inclusion/exclusion criteria: (1) Singleton pregnancies where RFM was reported for all observations; (2) reports included cases of stillbirth, fetal growth restriction (FGR) or new‐borns admitted to neonatal intensive care units (NICU admission); (3) participating women were aged between 16 and 50 years and within a gestational period of 28^+0^ to 41^+0^ weeks, and were able to give written informed consent; (4) fetuses had no congenital anomalies and there was no clinical indication for immediate delivery (e.g., pathological fetal heart rate trace), to provide further evidence for clinical decision making in uncertain cases, rather than in emergencies where immediate delivery is already clinically indicated; and (5) this was not their first contact with maternal services.

### 2.2 Data Collection Process

Authors of eligible studies were contacted by email and invited to provide the original dataset of their studies. Data from individual studies were collected at an individual participant level through mothers' case records during routine assessments (maternal demographics, details of the duration of RFM, and past obstetric and medical history), fetal heart rate trace (cardiotocograph) and biochemical testing (biomarkers measurement). Variable definitions and data‐coded instructions were provided by authors.

### 2.3 Data Items and IPD Integrity

Available explanatory variables were selected by searching for the same variables in each study. Individual participant data were checked by using exploratory data analysis, including identifying missing values and outliers, visualising distribution, correlation analysis and feature engineering (e.g., encoding, binning and combination). All IPD were then assembled in a single dataset, each observation labelled with a study‐specific identifier to denote its original source.

### 2.4 Risk of Bias Assessment Within Individuals and Across Studies

The Risk of Bias In Non‐randomised Studies—of Exposure (ROBINS‐E) tool [19] was applied to assess the risk of bias within individual studies. Egger's regression test was applied to assess the publication bias across studies.

### 2.5 Specification of Outcomes and Effect Measures

Adverse pregnancy outcome (APO) was defined as the occurrence of any of the following criteria: stillbirth, fetal growth restriction (FGR) (individualised birthweight centile ≤ 3rd centile) or NICU admission at term (gestation at delivery ≥ 37 weeks) (Table 2). Although preterm birth and small for gestational age infants (birthweight < 10th centile) were included in composite adverse outcomes in some individual studies, they were excluded from this analysis to maintain a narrow focus on the most severe complications, which are likely to demonstrate the most consistent associations with the risk factors under investigation within our study's framework. The estimated effects of risk factors on the outcome were reported using odds ratios (OR) and 95% confidence intervals (CI), *p* values and heterogeneity levels.

### 2.6 Synthesis Methods

Multiple imputation (MI) was employed to handle missing data after integrating individual datasets. Ten imputed datasets were generated for the combined data and compared by density plots. The subsequent models accounted for the potential bias introduced by the missingness in the original data by including the missing indicators [20]. Factors were selected on the basis of their consistent availability across all studies. Comparison between groups was by *t*‐test, Mann–Whitney test or chi‐squared/Fisher's exact test, as appropriate (0.05 level of significance). Multivariable logistic regression was used to assess the effects of primary exposures on APO, with each exposure adjusted for its specific set of potential confounders.

**FIGURE 1:**
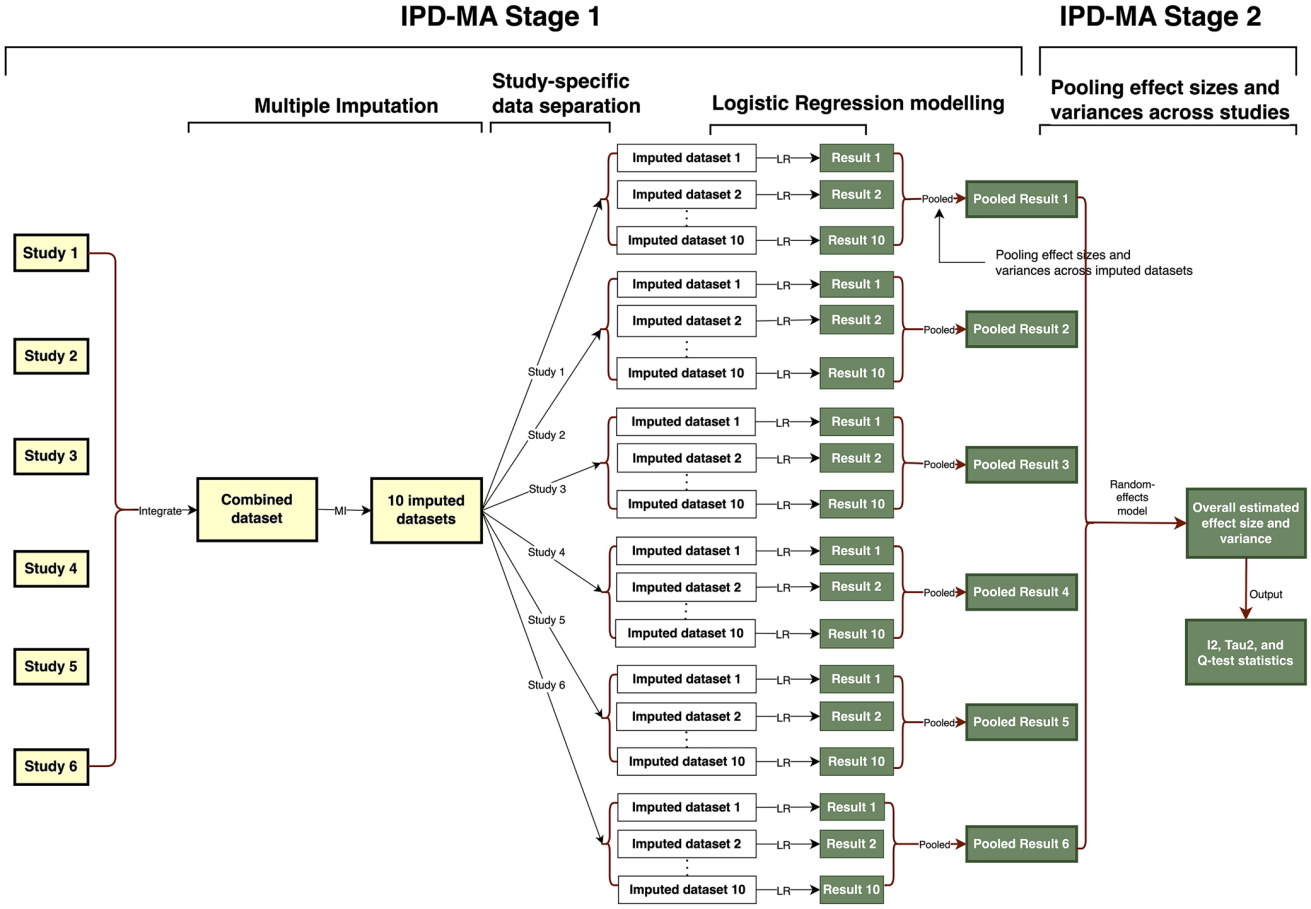
Flow chart of the two‐stage IPD meta‐analysis.

A two‐stage IPD‐MA [21, 22] was performed across six datasets (Figure 1). Stage 1 involved logistic regression on each study within each imputed dataset to obtain effect estimates and variances. At Stage 2, we combined the study‐specific effects using a random‐effects meta‐analysis model [21, 23, 24]. The between‐study variance in the random‐effects model was estimated using Tau‐squared (*τ* ^2^), whereas the *I* ^2^ statistic quantified the proportion of total variance in effect estimates that is attributable to heterogeneity. Cochran's *Q* test assessed whether there was significant heterogeneity among the effect sizes of the studies [25, 26, 27, 28]. Forest plots displayed the effect estimates for each study, along with their confidence intervals, allowing for exploration of variation across studies and visualisation of the pooled effect. Risk factors were identified by a significant association in multivariable models.

### 2.7 Additional Analyses

Sensitivity analyses were conducted to further validate the robustness of the main IPD‐MA. We repeated the procedures from the main IPD‐MA but excluded the intervention arms from the randomised controlled trials (RCTs) in Sensitivity Analysis 1. Additionally, we conducted Sensitivity Analysis 2 to investigate the impact of MI on the results by comparing it to the complete‐case analysis.

## 3 Results

### 3.1 Study Selection and IPD Obtained

Figure S1 shows the PRISMA 2020 flow diagram for study identification. Literature searches identified six studies from journal articles and two from unpublished research studies. Following requests, one author did not respond, and one was restricted from sharing data because of data protection regulations. Thus, this IPD‐MA was ultimately confined to IPD derived from six eligible UK studies, including published (*n* = 936) [2, 4, 5, 29] and two unpublished studies (*n* = 239). A PRISMA‐IPD checklist is provided in Table S1.

### 3.2 Study Characteristics

In total, 1175 singleton pregnancies with perceived RFM between 2009 and 2021 were included in this IPD‐MA (Table 1). Among these, 7.7% (90/1175) met the criteria for the APO (Table 2 and Figure S2) with the most common complication reported (4.6%) being fetal growth restriction. Four participants had more than one complication across all studies.

**TABLE 1:** Inclusion and exclusion criteria of participating studies.

Study	Study setting & recruitment period	Maternal age 16–50 years	Fetus without any congenital anomalies	No indication for immediate delivery	Singleton pregnancy	Not attending antenatal care for the first time	Not participating in another trial affecting delivery timing or method	Give informed consent
Study 1 [2] *N* = 305 (Prospective)	Manchester 08.2009–10.2010	✓	✓	✓	✓	✓	Not mentioned	✓
Study 2 [5] *N* = 296 (Prospective)	Manchester 01.2012–05.2014	✓	✓	✓	✓	Not mentioned	Not mentioned	✓
Study 3 *N* = 132 (Prospective)	Manchester 10.2016–03.2017	✓	✓	✓	✓	Not mentioned	Not mentioned	✓
Study 4 *N* = 107 (Prospective)	Leicester 01.2020–04.2021	✓	✓	✓	✓	Not mentioned	Not mentioned	✓
Study 5 [4] *N* = 119 (RCT)	Manchester 10.2011–08.2012	✓	✓	✓	✓	Not mentioned	Not mentioned	✓
Study 6 [27] *N* = 216 (RCT)	Multi‐centre study (Eight UK maternity units) 03.2017–01.2018	✓	✓	✓	✓	✓	✓	✓

**TABLE 2:** Outcome counts in individual studies and total.

Outcome	Study 1	Study 2	Study 3	Study 4	Study 5	Study 6	APO by outcome
Stillbirth	0 (0.0%)	1 (0.3%)	1 (0.8%)	0 (0.0%)	0 (0.0%)	0 (0.0%)	2 (0.2%)
Fetal growth restriction (birthweight < 3rd centile)	26 (8.5%)	13 (4.4%)	1 (0.8%)	9 (8.4%)	1 (0.8%)	4 (1.9%)	54 (4.6%)
NICU admission (> 37 weeks' gestation)	2 (0.7%)	9 (3%)	6 (4.5%)	6 (5.6%)	4 (3.4%)	11 (5.1%)	38 (3.2%)
Total by study (individual outcomes)	28 (9.2%)	23 (7.8%)	8 (6.1%)	15 (14.0%)	5 (4.2%)	15 (6.9%)	94 (8.0%)
Total by study (APO)	28 (9.2%)	22 (7.4%)	7 (5.3%)	14 (13.1%)	5 (4.2%)	14 (6.5%)	90 (7.7%)

*Note:* NB individual cases could have more than one adverse outcome, so the total adverse outcome count is greater than the composite measure (APO).

### 3.3 IPD Integrity

Twenty common explanatory variables were identified across the six studies (Table S3). In the combined dataset, the level of missingness ranged from 0.09% to 9.96%, with nearly 10% missing data for fetal heart rate assessment and approximately 7.5% missing for amniotic fluid index (Table S4).

### 3.4 Risk of Bias Within Studies

Four studies included in this IPD‐MA were rated as having an overall high risk of bias, driven predominantly by the post‐exposure interventions domain, where clinical decisions such as emergency Caesareans or inductions, influenced by exposure, likely attenuated observed associations between the factors and APO. Additional concerns were noted in the confounding domain across all studies, as none fully adjusted for unmeasured factors such as maternal health and socioeconomic status, potentially distorting associations. Bias from exposure measurement and outcome measurement was also noted, though typically at a lower level of concern (Table S5).

### 3.5 Risk of Bias Across Studies

Egger's regression tests yielded no *p* values less than a threshold of 0.05, indicating no significant evidence of publication bias across the studies incorporated in this IPD‐MA.

### 3.6 Results of Individual Studies and Syntheses

Comparisons between RFM pregnancies with and without APO are described in Table S2. RFM pregnancies with APO were more likely to report significant past medical history (PMH) (40% vs. 22%, *p* < 0.001) and had a higher rate of cigarette use (18% vs. 10%, *p* = 0.041). Additionally, fetal heart rate abnormalities were more prevalent in RFM pregnancies with APO (27% vs. 9%, *p* < 0.001); they also presented with a longer duration of RFM (48.0 vs. 30.0 h, *p* = 0.009) at an earlier stage of gestation (36.5 vs. 37.3 weeks, *p* = 0.016) and exhibited lower EFW centile (41.0 vs. 73.5, *p* < 0.001) compared to those without APO. There were no significant differences in maternal age, BMI, ethnicity, parity, systolic/diastolic blood pressure, alcohol use, past obstetric history, and ultrasound measure of blood flow (UAPI) and amniotic fluid (AFI) between participants with and without APO. There was no difference in APO prevalence between individual studies (Table S2).

**FIGURE 2:**
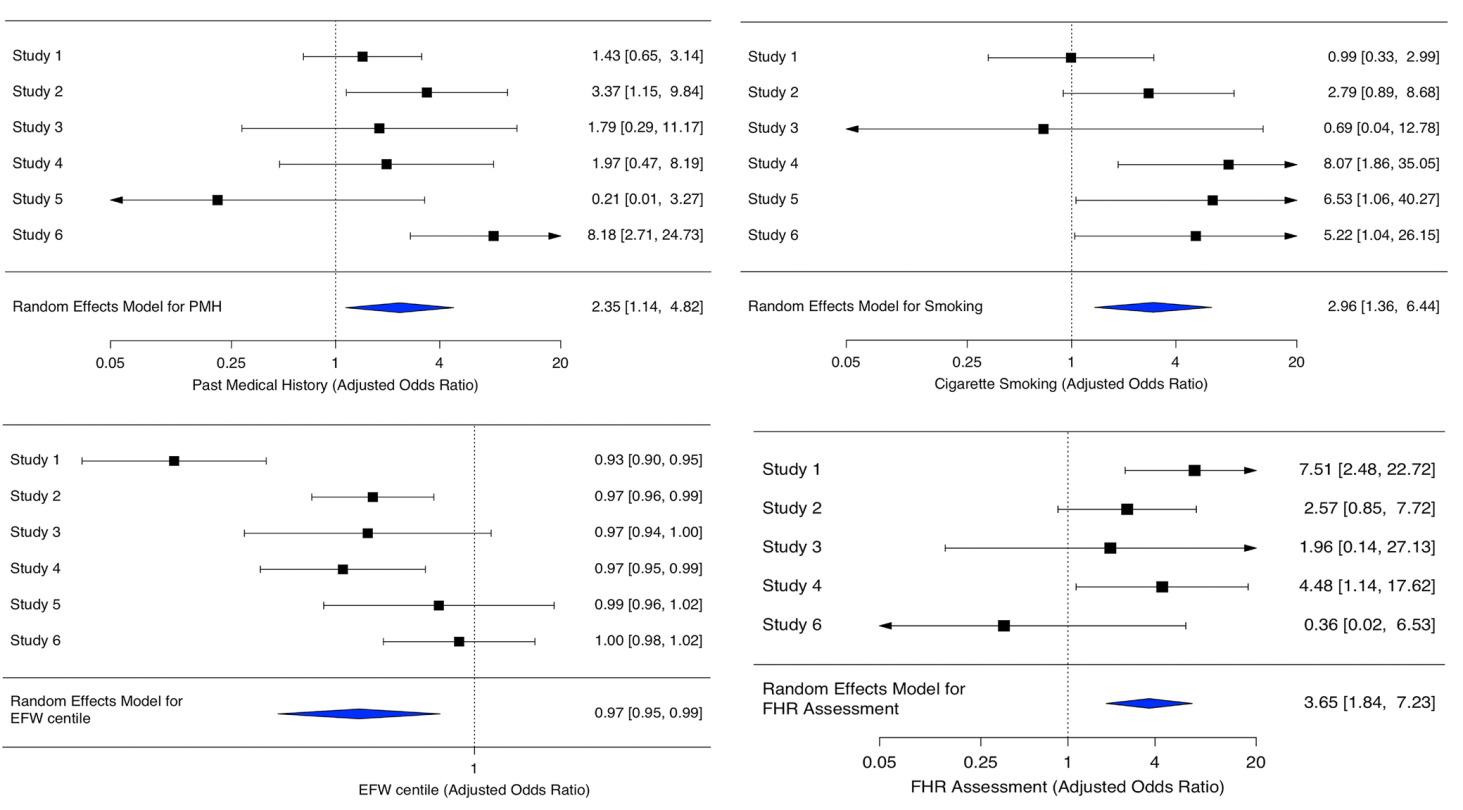
Forest plots of the effect sizes of abnormal fetal heart rate, EFW percentile, past medical history and cigarette smoking status on APO, alongside the pooled effect size across six studies.

In multivariable logistic regression models, having a past medical history (adjusted odds ratio [aOR] 2.35 [1.14, 4.82]) was a significant risk factor for APO, adjusting for maternal age (Table 3, Figure 2). PMH encompassed a broad range of conditions, including asthma, deep vein thrombosis (DVT), epilepsy, hypertension, hypothyroidism, obesity, polycystic ovary syndrome (PCOS), infertility and depression, among others (Table S6). Furthermore, the synthesised result from all studies showed that smokers (aOR 2.96 [1.36, 6.44]) and an abnormal fetal heart rate (aOR 3.65 [1.84, 7.23]) were associated with higher odds of experiencing APO after adjustment for maternal age, ethnicity and having a medical history. Additionally, for every unit increase in EFW centile at RFM presentation, the odds of experiencing APO decreased by 3% (aOR 0.97 [0.95, 0.99]) (Table 3, Figure 2).

**TABLE 3:** IPD meta‐analysis results (log ORs, adjusted ORs, *p* values, confidence intervals on OR scale and the results of heterogeneity check) of risk factors on the composite adverse pregnancy outcome (multivariable analysis).

Risk factor	Log‐OR	Adjusted OR	*p*	CI (OR scale)	*τ* ^2^	*I* ^2^ (%)	*Q*‐statistic *p*	Egger test *p*
Past medical history^a^	0.85	2.35	0.020	(1.14, 4.82)	0.3460	45.88	0.076	0.607
Cigarette smoking^b^	1.09	2.96	0.006	(1.36, 6.44)	0.3356	36.79	0.174	0.700
Abnormal fetal heart rate^b^	1.29	3.65	< 0.001	(1.84, 7.23)	0.0544	8.41	0.311	0.193
EFW percentile^c^	−0.03	0.97	0.005	(0.95, 0.99)	0.0005	80.74	< 0.001	0.811
Duration of RFM^d^	0.003	1.003	0.129	(0.999, 1.006)	0	0.00	0.633	0.987

^a^ Adjusted for maternal age.

^b^ Adjusted for maternal age, ethnicity and PMH.

^c^ Adjusted for maternal age, ethnicity, PMH and gestation at RFM presentation.

^d^ Adjusted for maternal age, BMI and EFW centile.

A high heterogeneity in EFW centile (*I* ^2^ = 80.74%, τ^2^ = 0.0005) was observed. The significant *Q*‐statistic *p* value (< 0.05) suggested that the observed variability in the effect sizes was the true differences between studies rather than sampling error or random variation. In contrast, cigarette smoking, PMH and abnormal fetal heart rate did not demonstrate significant heterogeneity in their effects on APO across studies (Table 3).

### 3.7 Additional Analyses

The results for PMH, EFW centile and abnormal fetal heart rate were consistent across all analyses, showing significant associations with APO. Cigarette smoking was significantly associated with APO, but this association became non‐significant when intervention arms were excluded (Table S8). Results from the complete‐case analyses closely aligned with those obtained from the imputed data (Tables S7 and S8). All sensitivity analyses and the main IPD‐MA consistently showed that the effect of EFW centile on the outcome has significant heterogeneity across individual studies (Table S8).

## 4 Discussion

### 4.1 Main Findings

This IPD‐MA identified maternal smoking status, PMH, abnormal fetal heart rate and EFW centile at RFM presentation as important risk factors for APO in women with RFM during the third trimester. Our findings provide new insights by reinforcing the significance of these risk factors in clinical practice, particularly in the presence of RFM.

These results are consistent with a previous systematic review which found an increase in abnormal fetal heart rate patterns among neonates with hypoxic–ischaemic encephalopathy, although the predictive ability was limited [30]. Additionally, certain pathological fetal heart rate patterns during labour were linked to increased NICU admissions [31]. These findings emphasise the importance of fetal heart rate assessment in high‐risk pregnancies, including after presentation with RFM. Maternal smoking has been consistently linked to placental dysfunction and fetal hypoxia [30], underscoring the critical need for continued surveillance of smoking behaviours among pregnant women to effectively reduce this risk. Additionally, the association between pre‐existing medical conditions and APO after RFM is supported by studies emphasising the impact of maternal comorbidities, particularly those affecting the cardiovascular and endocrine systems, on placental function and fetal well‐being [32]. Comprehensive antenatal care for women with PMH is crucial to mitigate potential risks. The association between low EFW centile and the increased odds of APO suggests that fetal growth monitoring is an important component of evaluation after adding RFM, as it could enable earlier intervention to mitigate adverse outcomes. This finding also aligns with the trends observed in Mlynarczyk et al. [33], which reported that neonates with sonographic‐estimated fetal weight below the 5th percentile experienced a higher rate of neonatal morbidity (31%) compared to those within the 5th to 9th percentile (13%). This finding further indicates that lower EFW centiles in women presenting with RFM in the third trimester correlate with increased risks [33].

### 4.2 Strengths

Unlike the traditional meta‐analyses that rely on aggregated data, IPD‐MA allows for the reanalysis of raw data across studies in a consistent way. This approach enables more precise data management and analysis:

1. Improved data consistency and harmonisation: We tailored outcome definitions to align with our research objectives—for example, defining fetal growth restriction as birth weight ≤ 3rd percentile, rather than the 10th centile as in previous studies. This approach enables the reanalysis of raw data across studies in a standardised and consistent manner to improve comparability [34]. Additionally, we applied multiple imputation across the entire IPD to ensure methodological consistency and reduce bias.
2. Accounted for between‐study heterogeneity: The random‐effects model quantified the heterogeneity and pooled estimates, providing more reliable insights into our clinical question. In this IPD‐MA, significant heterogeneity in the association between EFW percentile and APO suggests variability in the effect of EFW centile on the outcome across different settings or populations, rather than sampling error or random variation. Despite adjustments for maternal age, ethnicity, PMH and gestation at RFM presentation, the persistence of this heterogeneity may be attributable to several reasons, such as variations in the measurement techniques (e.g., ultrasound equipment, operator expertise and protocols) and timing of EFW assessments. Additionally, the populations studied may differ in genetic predispositions, lifestyle factors not accounted for, nutritional and socioeconomic status, all of which can influence fetal growth patterns and thus EFW centiles. Therefore, future work is needed to better understand the impact of these variables on fetal growth and pregnancy outcomes.
3. Enhanced sensitivity analysis: multiple sensitivity analyses were conducted to explore how different combinations of studies or data influenced the relationships between the risk factors and APO. In the main IPD‐MA, cigarette smoking was significantly associated with an increased risk of APO. However, this association was not significant in the sensitivity analysis that excluded the intervention arms from RCTs, leaving only the cohort studies and the control arms. This change implies that the variation in smoking status between two arms may have contributed to the initial significant effect. In the main analysis, the smoking rates varied more widely (from 6.4% to 11.7%), which could have introduced greater variability in the data, making the relationship between smoking and adverse outcomes more detectable. After removing the intervention arms, the remaining sample became more homogeneous in terms of smoking distribution (around 8.5% to 9.4%), which reduced the variability and, consequently, the statistical significance of smoking status as a risk factor for APO.

### 4.3 Limitations

#### 4.3.1 Search Strategy

Individual studies relevant to RFM were selected on the basis of their relevance and data availability, with all included studies meeting predefined criteria. Efforts were made to extend the scope of this analysis by reaching out to authors of two additional studies; it is possible that some relevant studies were omitted from the analysis. As a result, the generalisability of our findings beyond the included settings may be limited. Nonetheless, including diverse maternity units enhances their applicability within the UK context. Future research incorporating a comprehensive search strategy will be valuable in further broadening the evidence base.

#### 4.3.2 Risk of Bias Within Individual Studies

Although the studies provided relevant data, the high risk of bias, particularly from post‐exposure interventions and confounding, might mask the true exposure–outcome relationships, suggesting that the results must be interpreted cautiously. For instance, clinical interventions (e.g., emergency Caesarean births and induction of labour) could have introduced significant bias by likely mitigating adverse outcomes. Additionally, missing data across individual datasets necessitated the use of multiple imputation, assuming data were missing at random (MAR) or missing completely at random (MCAR) [35]. Although complete‐case analysis results aligned closely with those from the main analysis, the wider confidence intervals in the complete‐case analysis reflect a reduced sample size and potential underlying biases. Moreover, comparing the stillbirth rate in high‐income countries with global trends highlights the need for more inclusive datasets, particularly from low‐ and middle‐income countries, where the majority of stillbirths occur [36]. Expanding such datasets could yield more generalisable results and help ensure the robustness of our findings.

#### 4.3.3 Outcome Definition

Preterm births (including both spontaneous and iatrogenic, regardless of medical indication) were included in the dataset but were not classified as an APO unless they met the composite definition (stillbirth, FGR—birthweight ≤ 3rd centile or NICU admission at birth gestation ≥ 37 weeks). This approach ensured a focus on the most severe complications. However, iatrogenic preterm birth, even if not an APO, may have influenced the observed associations between risk factors and APO. If high‐risk pregnancies were delivered preterm before reaching APO criteria, some severe cases may have been shifted out of the APO group, potentially affecting the observed associations. This aligns with challenges discussed by Gordijn et al., highlighting how outcome definitions can impact risk factor analysis [37]. To address this, a further sensitivity analysis would be beneficial to assess whether iatrogenic preterm birth influenced the estimated effects of risk factors on APO.

#### 4.3.4 Population—Inclusion Criteria, Ethnicity and Behavioural

Cases requiring immediate delivery due to an obvious clinical need were excluded, which aimed to ensure that the analysis focuses on pregnancies where ongoing RFM is the primary concern. However, excluding these cases may have omitted more severe consequences of RFM, thereby underestimating the full spectrum of risks associated with RFM on the outcomes. To address this, future studies could stratify immediate delivery cases as a separate group or conduct sensitivity analyses to assess the impact of their exclusion.

Most participants in our IPD were White (85%), suggesting potential selection bias within and across studies, as non‐White women may be less likely to seek obstetric care for RFM [38, 39]. Similarly, no participants in Studies 3 and 4 reported alcohol consumption during pregnancy, which is inconsistent with the contemporaneous Midlands and North of England Stillbirth Study, where a 6.1% incidence of alcohol consumption was reported [40]. This discrepancy raises concerns about potential selection bias in Studies 3 and 4, where zero alcohol consumption may not accurately reflect broader population behaviours.

#### 4.3.5 Adverse Events

Stillbirth is uncommon in the UK, with a recorded rate of 0.35% in 2021, compared with a global average of 1.39% in the same year [41, 42]. This lower incidence may influence the detection of associations between certain conditions and adverse pregnancy outcomes in our study. Future research should include more geographically diverse data. For instance, an IPD‐MA by Thompson et al. [1] incorporated data from New Zealand, Australia, the UK, and an internet‐based study based out of the USA. Including data from regions with higher rates of APO, such as low‐income and developing countries, would ensure a more representative number of events. This diversification will increase the sample size and enhance the statistical power of future analyses, yielding more widely applicable results.

#### 4.3.6 Past Medical History

This IPD‐MA did not delve into the impact of specific prior health conditions—such as significant cardiovascular diseases, reproductive health issues or other conditions that may contribute to the impact on adverse pregnancy outcomes. This highlights a critical area for future research that could provide more comprehensive insights into the impact of specific medical history on adverse outcomes.

#### 4.3.7 Consideration of Current Obstetric Conditions

Low EFW, hypertensive diseases of pregnancy or gestational diabetes often require specific interventions according to protocols that may potentially influence management decisions and outcomes. Future research could benefit from investigating a subgroup of pregnancies without predefined obstetric conditions at RFM presentation to better understand the isolated effects of risk factors on adverse outcomes in RFM pregnancies.

#### 4.3.8 Study Design of Individual Studies

Including both cohorts and RCTs could introduce methodological heterogeneity, complicating result interpretation. For example, the discrepancy of smoking's effect when included and excluded RCTs highlights the critical role of study design and confounding control. In Study 6, a blood test measuring the sFlt‐1/PLGF ratio, a biomarker indicative of placental function, informed clinical decisions about early delivery or levels of care [29]. The outcomes modelled in RCTs occur post‐intervention, which could influence results differently than observational studies where no interventions are applied. Future meta‐analyses should consider focusing on similar designs, such as exclusively RCTs or cohort studies, to help ensure comparability in the meta‐analysis.

### 4.4 Interpretation and Implication

Reduced fetal movements are a common indication of adverse pregnancy outcomes. This impacts medical resources through increased medical interventions and potentially raises other adverse effects on newborn health [43, 44]. This IPD‐MA provides a foundation for shaping clinical guidelines and future research in RFM and APO. It emphasises the importance of reducing smoking during pregnancy, improving the management of pre‐existing medical conditions, and ensuring adequate monitoring of fetal growth and fetal heart rate after presentation with RFM to mitigate risks. Although there is an association between the assessment of the fetal heart rate (by cardiotocography) and ultrasound EFW and APO, the increased odds of APO remain comparatively modest. Other assessments of fetal well‐being such as the cerebroplacental ratio have been evaluated to determine whether they can reduce morbidity without increasing rates of intervention [45]. We anticipate the factors that are significantly associated with adverse pregnancy outcomes can guide a more personalised approach to ensure appropriate interventions for women presenting with subjectively perceived reduced fetal activity, balancing timely intervention with the prevention of unnecessary procedures.

## 5 Conclusion

Maternal smoking status, pre‐existing medical conditions and abnormal fetal heart rate showed the strongest significant associations with APO in RFM pregnancies. The EFW centile also demonstrated a significant association with the outcome, although clear heterogeneity in EFW centile may reflect differences in quality and potential recording biases across individual studies. Optimising data collection procedures is crucial to ensure that comparable data are collected in studies of RFM, avoiding missingness in data and expanding datasets to include diverse populations will enhance the robustness and generalisability of results in future IPD meta‐analyses. Applying the core outcome set for studies of RFM [34] will ensure that future studies will collect important information about factors associated with APO after maternal presentation with RFM.

## Author Contributions

V.P. and A.H. conceived the IPD‐MA, provided the comments and refined the paper. A.H. provided the raw data. Y.L. pre‐processed and analysed the data and wrote the draft manuscript.

## Ethics Statement

Individual studies were approved by the Human Research Authority (FEMINA1‐08/H1011/83; FEMINA2‐11/NW/0650; FEMINA3‐16/NW/0481; ReMIT‐11/NW/0664, ReMIT‐2‐17/NW/0014).

## Conflicts of Interest

The authors declare no conflicts of interest.

## Supporting information


**Figure S1.** PRISMA flow diagram for study identification.
**Figure S2.** Sunburst chart showing the distribution of participants and cases of APO across six studies.
**Table S1.** PRISMA‐IPD Checklist of items to include when reporting a systematic review and meta‐analysis of individual participant data (IPD).
**Table S2.** The characteristic table on each level in APO and individual outcomes (The baseline characteristics include demographic data, maternal physical information, lifestyle behaviours data during this pregnancy (e.g., smoking status and alcohol consumption), medical and obstetric history, and current pregnancy and obstetric data).
**Table S3.** Common variables across six studies.
**Table S4.** The percentages of missingness in variables.
**Table S5.** Results of risk of bias assessment in individual studies using ROBINS‐E.
**Table S6.** Past medical history reported by participants in each study.
**Table S7.** Complete‐case IPD Meta‐analysis results (log ORs, ORs, *p* values, confidence intervals on OR scale and the results of heterogeneity check) of risk factors on the Adverse Pregnancy Outcome (Multivariable Analysis).
**Table S8.** Results of sensitivity analyses by excluding the intervention arm from RCTs (adjusted ORs, 95% confidence intervals on OR scale, *p* values and the *Q*‐statistic *p* value of heterogeneity check) of risk factors on the Adverse Pregnancy Outcome (based on different sensitivity analyses and data used).

## Data Availability

The data that support the findings of this study are available from corresponding authors upon reasonable request. The data are not publicly available due to privacy or ethical restrictions.
