# Supplementary material for: Risk Factors for Adverse Pregnancy Outcomes in Reduced Fetal Movement: An IPD Meta‐Analysis
Source: BJOG. 2025 Mar 17;132(7):1000–9. doi: 10.1111/1471-0528.18132 (PMC12051240; doi:10.1111/1471-0528.18132)
Supplement: Supplementary file 1 — Figure S1. PRISMA flow diagram for study identification. Figure S2. Sunburst chart showing the distribution of participants and cases of APO across six studies. Table S1. PRISMA‐IPD Checklist of items to include when reporting a systematic review and meta‐analysis of individual participant data (IPD). Table S2. The characteristic table on each level in APO and individual outcomes (The baseline characteristics include demographic data, maternal physical information, lifestyle behaviours data during this pregnancy (e.g., smoking status and alcohol consumption), medical and obstetric history, and current pregnancy and obstetric data). Table S3. Common variables across six studies. Table S4. The percentages of missingness in variables. Table S5. Results of risk of bias assessment in individual studies using ROBINS‐E. Table S6. Past medical history reported by participants in each study. Table S7. Complete‐case IPD Meta‐analysis results (log ORs, ORs, p values, confidence intervals on OR scale and the results of heterogeneity check) of risk factors on the Adverse Pregnancy Outcome (Multivariable Analysis). Table S8. Results of sensitivity analyses by excluding the intervention arm from RCTs (adjusted ORs, 95% confidence intervals on OR scale, p values and the Q‐statistic p value of heterogeneity check) of risk factors on the Adverse Pregnancy Outcome (based on different sensitivity analyses and data used). [file BJO-132-1000-s001.docx]

**Supplementary information**

*Table S1: PRISMA-IPD Checklist of items to include when reporting a systematic review and meta-analysis of individual participant data (IPD)*

| **PRISMA-IPD**  **Section/topic** | **Item No** | **Checklist item** | **Reported on page** |
| --- | --- | --- | --- |
| **Title** | | | |
| Title | 1 | Identify the report as a systematic review and meta-analysis of individual participant data. | 1 |
| **Abstract** | | | |
| Structured summary | 2 | Provide a structured summary including as applicable: | 2 |
|  |  | **Background**: state research question and main objectives, with information on participants, interventions, comparators and outcomes. |  |
|  |  | **Methods**: report eligibility criteria; data sources including dates of last bibliographic search or elicitation, noting that IPD were sought; methods of assessing risk of bias. |  |
|  |  | **Results**: provide number and type of studies and participants identified and number (%) obtained; summary effect estimates for main outcomes (benefits and harms) with confidence intervals and measures of statistical heterogeneity. Describe the direction and size of summary effects in terms meaningful to those who would put findings into practice. |  |
|  |  | **Discussion:** state main strengths and limitations of the evidence, general interpretation of the results and any important implications. |  |
|  |  | **Other:** report primary funding source, registration number and registry name for the systematic review and IPD meta-analysis. |  |
| **Introduction** | | | |
| Rationale | 3 | Describe the rationale for the review in the context of what is already known. | N/A |
| Objectives | 4 | Provide an explicit statement of the questions being addressed with reference, as applicable, to participants, interventions, comparisons, outcomes and study design (PICOS). Include any hypotheses that relate to particular types of participant-level subgroups. | 4 |
| **Methods** | | | |
| Protocol and registration | 5 | Indicate if a protocol exists and where it can be accessed. If available, provide registration information including registration number and registry name. Provide publication details, if applicable. | N/A |
| Eligibility criteria | 6 | Specify inclusion and exclusion criteria including those relating to participants, interventions, comparisons, outcomes, study design and characteristics (e.g. years when conducted, required minimum follow-up). Note whether these were applied at the study or individual level i.e. whether eligible participants were included (and ineligible participants excluded) from a study that included a wider population than specified by the review inclusion criteria. The rationale for the criteria should be stated. | 5 |
| Identifying studies - information sources | 7 | Describe all methods of identifying published and unpublished studies including, as applicable: which bibliographic databases were searched with dates of coverage; details of any hand searching including of conference proceedings; use of study registers and agency or company databases; contact with the original research team and experts in the field; open adverts and surveys. Give the date of last search or elicitation. | 5 |
| Identifying studies - search | 8 | Present the full electronic search strategy for at least one database, including any limits used, such that it could be repeated. | 5 |
| Study selection processes | 9 | State the process for determining which studies were eligible for inclusion. | 5 |
| Data collection processes | 10 | Describe how IPD were requested, collected and managed, including any processes for querying and confirming data with investigators. If IPD were not sought from any eligible study, the reason for this should be stated (for each such study). | 5 |
|  |  | If applicable, describe how any studies for which IPD were not available were dealt with. This should include whether, how and what aggregate data were sought or extracted from study reports and publications (such as extracting data independently in duplicate) and any processes for obtaining and confirming these data with investigators. |  |
| Data items | 11 | Describe how the information and variables to be collected were chosen. List and define all study level and participant level data that were sought, including baseline and follow-up information. If applicable, describe methods of standardising or translating variables within the IPD datasets to ensure common scales or measurements across studies. | 6 |
| IPD integrity | A1 | Describe what aspects of IPD were subject to data checking (such as sequence generation, data consistency and completeness, baseline imbalance) and how this was done. | 6 |
| Risk of bias assessment in individual studies. | 12 | Describe methods used to assess risk of bias in the individual studies and whether this was applied separately for each outcome. If applicable, describe how findings of IPD checking were used to inform the assessment. Report if and how risk of bias assessment was used in any data synthesis. | 6 |
| Specification of outcomes and effect measures | 13 | State all treatment comparisons of interests. State all outcomes addressed and define them in detail. State whether they were pre-specified for the review and, if applicable, whether they were primary/main or secondary/additional outcomes. Give the principal measures of effect (such as risk ratio, hazard ratio, difference in means) used for each outcome. | 6 |
| Synthesis methods | 14 | Describe the meta-analysis methods used to synthesise IPD. Specify any statistical methods and models used. Issues should include (but are not restricted to):   - Use of a one-stage or two-stage approach. - How effect estimates were generated separately within each study and combined across studies (where applicable). - Specification of one-stage models (where applicable) including how clustering of patients within studies was accounted for. - Use of fixed or random effects models and any other model assumptions, such as proportional hazards. - How (summary) survival curves were generated (where applicable). - Methods for quantifying statistical heterogeneity (such as I^2^ and t^2^). - How studies providing IPD and not providing IPD were analysed together (where applicable). - How missing data within the IPD were dealt with (where applicable). | 7 |
| Exploration of variation in effects | A2 | If applicable, describe any methods used to explore variation in effects by study or participant level characteristics (such as estimation of interactions between effect and covariates). State all participant-level characteristics that were analysed as potential effect modifiers, and whether these were pre-specified. | 7 |
| Risk of bias across studies | 15 | Specify any assessment of risk of bias relating to the accumulated body of evidence, including any pertaining to not obtaining IPD for particular studies, outcomes or other variables. | 6 |
| Additional analyses | 16 | Describe methods of any additional analyses, including sensitivity analyses. State which of these were pre-specified. | 7 |
| Results | | | |
| Study selection and IPD obtained | 17 | Give numbers of studies screened, assessed for eligibility, and included in the systematic review with reasons for exclusions at each stage. Indicate the number of studies and participants for which IPD were sought and for which IPD were obtained. For those studies where IPD were not available, give the numbers of studies and participants for which aggregate data were available. Report reasons for non-availability of IPD. Include a flow diagram. | 8 |
| Study characteristics | 18 | For each study, present information on key study and participant characteristics (such as description of interventions, numbers of participants, demographic data, unavailability of outcomes, funding source, and if applicable duration of follow-up). Provide (main) citations for each study. Where applicable, also report similar study characteristics for any studies not providing IPD. | 8 |
| IPD integrity | A3 | Report any important issues identified in checking IPD or state that there were none. | 8 |
| Risk of bias within studies | 19 | Present data on risk of bias assessments. If applicable, describe whether data checking led to the up-weighting or down-weighting of these assessments. Consider how any potential bias impacts on the robustness of meta-analysis conclusions. | 8 |
| Results of individual studies | 20 | For each comparison and for each main outcome (benefit or harm), for each individual study report the number of eligible participants for which data were obtained and show simple summary data for each intervention group (including, where applicable, the number of events), effect estimates and confidence intervals. These may be tabulated or included on a forest plot. | 9 |
| Results of syntheses | 21 | Present summary effects for each meta-analysis undertaken, including confidence intervals and measures of statistical heterogeneity. State whether the analysis was pre-specified, and report the numbers of studies and participants and, where applicable, the number of events on which it is based. | 9 |
|  |  | When exploring variation in effects due to patient or study characteristics, present summary interaction estimates for each characteristic examined, including confidence intervals and measures of statistical heterogeneity. State whether the analysis was pre-specified. State whether any interaction is consistent across trials. |  |
|  |  | Provide a description of the direction and size of effect in terms meaningful to those who would put findings into practice. |  |
| Risk of bias across studies | 22 | Present results of any assessment of risk of bias relating to the accumulated body of evidence, including any pertaining to the availability and representativeness of available studies, outcomes or other variables. | 9 |
| Additional analyses | 23 | Give results of any additional analyses (e.g. sensitivity analyses). If applicable, this should also include any analyses that incorporate aggregate data for studies that do not have IPD. If applicable, summarise the main meta-analysis results following the inclusion or exclusion of studies for which IPD were not available. | 10 |
| **Discussion** | | | |
| Summary of evidence | 24 | Summarise the main findings, including the strength of evidence for each main outcome. | 11 |
| Strengths and limitations | 25 | Discuss any important strengths and limitations of the evidence including the benefits of access to IPD and any limitations arising from IPD that were not available. | 12 |
| Conclusions | 26 | Provide a general interpretation of the findings in the context of other evidence. | 17 |
| Implications | A4 | Consider relevance to key groups (such as policy makers, service providers and service users). Consider implications for future research. | 16 |
| **Funding** | | | |
| Funding | 27 | Describe sources of funding and other support (such as supply of IPD), and the role in the systematic review of those providing such support. | 19 |

*Table S2: The characteristic table on each level in APO and individual outcomes (The baseline characteristics include demographic data, maternal physical information, lifestyle behaviours data during this pregnancy (e.g., smoking status and alcohol consumption), medical and obstetric history, and current pregnancy and obstetric data.*

| Adverse Pregnancy Outcome | Normal (n=1085) | Adverse (n=90) | P-value |
| --- | --- | --- | --- |
| Individual Studies |  |  | 0.111 |
| Study 1 | 277 (25.5%) | 28 (31.1%) |  |
| Study 2 | 274 (25.3%) | 22 (24.4%) |  |
| Study 3 | 125 (11.5%) | 7 (7.8%) |  |
| Study 4 | 93 (8.6%) | 14 (15.6%) |  |
| Study 5 | 114 (10.5%) | 5 (5.6%) |  |
| Study 6 | 202 (18.6%) | 14 (15.6%) |  |
| Maternal Characteristics | | | |
| Maternal Age (Years) | 28 (24.0-32.0) | 29.0 (25.0-34.0) | 0.121 |
| Ethnicity |  |  | 0.882 |
| European | 731(67.4%) | 61 (67.8%) |  |
| Asian | 150 (13.8%) | 16 (17.8%) |  |
| African | 106 (9.8%) | 8 (8.9%) |  |
| Middle/Far Eastern | 49 (4.5%) | 3 (3.3%) |  |
| Mixed/others | 36 (3.3%) | 2 (2.2%) |  |
| Unknown | 13 (1.2%) | 0 (0.0%) |  |
| BMI (kg/m2) | 25.8 (22.8-30.1) | 25.5 (23.4-30.2) | 0.973 |
| SBP (mmHg) | 110.0 (100.0-120.0) | 111.5 (107.0-121.0) | 0.093 |
| DBP (mmHg) | 63.0 (60.0-72.0) | 66.0 (60.0-72.8) | 0.689 |
| Parity |  |  | 0.882 |
| 0 | 563 (51.9%) | 43 (48%) |  |
| 1 | 314 (28.9%) | 30 (33%) |  |
| 2 | 128 (11.8%) | 13 (14%) |  |
| 3 | 54 (5.0%) | 3 (3.3%) |  |
| 4+ | 25 (2.3%) | 1 (1.1%) |  |
| Unknown | 1 (0.1%) | 0 (0.0%) |  |
| Lifestyle Behaviour | | | |
| Current use of cigarettes | 111 (10%) | 16 (18%) | 0.041 |
| Current use of alcohol | 16 (1.5%) | 1 (1.1%) | 1.000 |
| Medical and Obstetric History | | | |
| Have Past Medical History | 243 (22%) | 36 (40%) | <0.001 |
| Have Past Obstetric History of Complications | 254 (23%) | 24 (27%) | 0.569 |
| RFM Episode Characteristic | | | |
| Gestation of RFM Presentation | 37.3 (34.1-39.0) | 36.5 (33.4-38.1) | 0.016 |
| Duration of RFM (hours) | 30.0 (15.0-48.0) | 48.0 (24.0-72.0) | 0.009 |
| Have Absent Fetal Movement | 412 (39%) | 34 (39%) | 1.000 |
| Abnormal Fetal Heart Rate | 88 (9.0%) | 22 (27%) | <0.001 |
| Estimated Fetal Weight Centile | 73.6 (51.2-87.5) | 41.1 (18.4-81.9) | <0.001 |
| AFI below 10th centile | 186 (19%) | 22 (25%) | 0.168 |
| AFI above 90th centile | 47 (4.7%) | 5 (5.7%) | 0.601 |
| UAPI above 95th centile | 38 (3.6%) | 7 (8.0%) | 0.076 |
| UAPI above 97.5th centile | 15 (1.4%) | 3 (3.4%) | 0.154 |
| Birth Outcome | | | |
| Birth Gestation | 39.7 (38.7-40.6) | 38.6 (37.3-40.4) | <0.001 |
| Fetal Sex (exclude Study 2) |  |  | 0.517 |
| Female | 385 (47.5%) | 29 (42.6%) |  |
| Male | 425 (52.4%) | 39 (57.4%) |  |
| Unknown | 1 (0.1%) | 0 (0.0 %) |  |
| Continuous values are median (1^st^ Qu – 3^rd^ Qu) or n (%), compared by a t-test for normally distributed data or Shapiro-Wilk test for non-normally distributed data.  Categorical data are expressed as number (%), compared by chi-squared test when all expected cell frequencies≥5 or Fisher’s Exact Test when any expected frequency<5.  Statistical significance was set at the level of p<0.05.  APO = adverse pregnancy outcome. FGR = fetal growth restriction. NICU = neonatal intensive care unit. BMI = body mass index.  SBP = systolic blood pressure. DBP = diastolic blood pressure. RFM = reduced fetal movement. AFI = amniotic fluid index.  UAPI = umbilical artery pulsatility index. | | | |

*Table S3: Common variables across 6 studies*

| Classification | Variables |
| --- | --- |
| Cluster variable | Study (Numeric, 1~6) - Which study the observation comes from |
| Demographic information | Maternal age (Numeric, 16~46) |
|  | Ethnicity (European/Asian/African/Middle and far eastern/Mixed and others) |
| Maternal physical characteristics | Body Mass Index (BMI) (Numeric) = weight (kg) / height2 (m2) |
|  | Systolic BP (mmHg) (Numeric) |
|  | Diastolic BP (mmHg) (Numeric) |
| Lifestyle behaviours data during this pregnancy | Current use of cigarettes (yes/no) |
|  | Current use of alcohol (yes/no) |
| Medical and obstetric history | Past medical history (yes/no) |
|  | Past obstetric history (yes/no) |
| Pregnancy and obstetric data | Parity (Numeric) - the number of previous pregnancies exceeding 24 weeks gestation |
|  | Gestation when presenting with RFM (Numeric) |
|  | Duration of RFM (hours) (Numeric) |
|  | Whether the participant experienced an absence of fetal movements (yes/no) |
|  | Fetal heart rate assessment on cardiotocography at the time of presentation with RFM (normal/abnormal), classified as normal if:  -The baseline rate was 110-160 bpm  -The variability was greater than 5 bpm  -Acceleration present  -No deceleration |
|  | Estimated fetal weight centile (Numeric) |
|  | Amniotic fluid index below the 10th percentile of reference value (yes/no) |
|  | Amniotic fluid index above the 90th percentile of reference value (yes/no) |
|  | Pulsatility index of the umbilical artery above the 95^th^ percentile of reference value (yes/no) |
|  | Pulsatility index of the umbilical artery above the 97.5^th^ percentile of reference value (yes/no) |

*Table S4: The percentages of missingness in variables*

| Variable | Missingness (%) |
| --- | --- |
| Fetal Heart Rate Assessment  (derived from Fetal Baseline Heart Rate, Accelerations, Decelerations, Variability) | 9.957 |
| Amniotic Fluid Index (AFI) | 7.489 |
| Systolic BP | 7.149 |
| Diastolic BP | 6.809 |
| Duration of RFM | 3.660 |
| Absence of Fetal Movement | 2.894 |
| Pulsatility Index of Umbilical Artery (UAPI) | 2.298 |
| Fetal Growth Restriction (birthweight < 3^rd^ centile) | 2.213 |
| Ethnicity | 1.106 |
| Stillbirth | 1.021 |
| Admitted to NICU (> 37 weeks' gestation) | 0.936 |
| Estimated Fetal Weight Percentile | 0.851 |
| Parity (Number of previous pregnancies exceeding 24 weeks’ gestation) | 0.851 |
| Maternal age | 0.170 |
| Alcohol Consumption | 0.085 |

*Table S5: Results of risk of bias assessment in individual studies using ROBINS-E*

| Study | Confounding | Selection Bias | Exposure Measurement | Post-Exposure Interventions | Missing Data | Outcome Measurement | Reporting Bias | Overall Risk of Bias |
| --- | --- | --- | --- | --- | --- | --- | --- | --- |
| Dutton et al., 2012 | Some concerns | Low | Some concern | High | Low | Low | Some concern | High, predicted towards null |
| Higgins et al., 2018 | Some concern | Low | Low | High | Some concern | Low | Some concern | High, predicted towards null |
| Heazell et al., 2013 | Some concern | Low | Some concern | High | Low | Low | Some concern | High, predicted towards null |
| Armstrong-Buisseret et al., 2020 | Some concern | Low | Some concern | High | Some concern | Low | Some concern | High, predicted towards null |
| *Judgment based on all domains and the signalling questions provided in the ROBINS-E guidelines.^15^ | | | | | | | | |

*Table S6: Past medical history reported by participants in each study*

| Column1 | Study 1 | Study 2 | Study 3 | Study 4 | Study 5 | Study 6 |
| --- | --- | --- | --- | --- | --- | --- |
| Respiratory Conditions | Asthma, Chronic Severe Asthma | The data in study 2 (Femina 2) didn't specify the types of medical history but has a variable that illustrates whether the individual had past medical history. |  | Asthma, R Lung Pulmonary Embolism | Asthma |  |
| Cardiovascular Conditions | Deep Vein Thrombosis (DVT), Raised blood Pressure, Hypertension, Palpitations, Mild Pulmonary Stenosis, Heart Murmur |  | Mitral Valve Disease | Ablation for AVNRT slow pathway, Arrhythmia - Supraventricular Tachycardia, Sinus Tachycardia, | Deep Vein Thrombosis (DVT), Mild heart murmur, Mitral Valve Regurgitation | Cardiovascular Condition x 3, High Blood Pressure x 4 |
| Endocrine/Metabolic Disorders | Hypothyroidism, Hyperthyroidism, Diabetes, Obesity, Vitamin D Deficiency |  | Hypothyroidism, HYPOPHOSPHATE MIC RICKETS | Hypothyroidism, Thyroid Gland Removal | Hypothyroidism, Prolactinaemia, High BMI | Diabetes x 2, Thyrd x 7 |
| Gastrointestinal Conditions | Appendicectomy, Cronhn's Disease, Cholecystectomy, Gastric Ulcer |  | Chronic Constipation, Coeliac's Disease, Crohn's Disease | Gallbladder Removal, Appendix, Appendicectomy, Gastric Sleeve Surgery, Chronic Cholestatic hepatitis, irritable bowel syndrome (IBS), Celiac Disease, Cholecystectomy, Gastroesophageal Reflux Disease (GORD), Hiatal Hernia, Gall Stones | Cronhn's Disease, IBS (irritable bowel syndrome), Duodenal ulcer, Gastric Bypass | Gastp x 3 |
| Neurological Conditions | Epilepsy, Migraine, Pituatary Adenoma, Squint, Glaucoma |  | Epilepsy | Symphysis Pubis Dysfunction (SPD), Pseudoseizures, Epilepsy, Childhood Seizures, Squint Repair, Pseudoseizures, Seizures - due to chemotherapy | Dystonic Torticollis, Chiari Malformation Required, Migraines Under Neurologist Care, Severe Dyslexia |  |
| Musculoskeletal Disorders | Scoliosis, Congenital Hip Dislocation, Osteopenia |  |  | Shoulder Operation, Knee Operation, Right Hip Awaiting Operation, Bunions Removed, Fibromyalgia, Hip Replacement, Osteoarthritis, Ganglion Cyst |  |  |
| Mental Health Issues | Depression, Anxiety, Bipolar Disorder, Cognitive Behavior Therapy |  |  | Bipolar Disorder, Depression, Personality Disorder, Eating Disorder, Anxiety, Post Natal Depression, Attention Deficit Hyperactivity Disorder (ADHD), Anxiety, Emotionally Unstable Personality Disorder (EUPD) | Depression (various mentions, including severe and with medications), Severe depression sectioned under mental health act, Eating disorders | Mental Health Issues x 10 |
| Renal Urological Conditions | Nephrectomy, Recurrent UTIs, Hydronephrosis |  | Congenital Extra Urethra, Recurrent UTIs |  |  | Renal Urological Condition x 1 |
| Hematological Disorders | Anti-phospholipid Syndrome, Clotting Disorder, Sickle Cell Disease, Pernicious Anaemia, Thalassaemia, Von Willebrand Disease with Thrombocytopenia |  | Low Platelets, ITP (Immune Thrombocytopenic Purpura), Factor V Leiden | B12 Deficiency, Low Platelets, Anemia, Cryofibrinogen Anemia | Anti-phospholipid Syndrome, Type 1 Von Willebrand's disease, Thrombocytopenia, Haemachromastasis, Idiopathic Thrombocytopenic |  |
| Reproductive Health Issues | Polycystic Ovarian Syndrome (PCOS), Endometriosis, Infertility, Fibroid Embolisation, Cervical Cytology Abnormal |  | Polycystic Ovarian Syndrome (PCOS) | Polycystic Ovarian Syndrome (PCOS), Infertility, Instrauterine Insemination, Endometriosis, Endometriosis Ablation, Pelvic Congestion Syndrome, Pelvic Inflammatory Disease (PID), Fallopian Tube Removal, Cervical Cautery Colposcopy, Ovarian Cyctectomy, Cyst on the left ovaian, Female Genital Mutilation (FGM), Dilation and Curettage (D&C), Pre-eclampsia, Hysteroscopy, Laparpscopy, Colposcopy, CF Carrier, Previous C/S, Emergency C/S, Abnormal Cervical Smears | Poist Coital Tear Requiring Transfusions, Infunibulation and Repair, Removal of a fallopian tube following an ectopic pregnancy, Uterine Prolapse |  |
| Infections | Hepatitis B, Hepatitis E, Genital Herpes, Swine Flu |  | Polio Infection | Group B Streptococcus (GBS) Positive, Recurrent Renal infections, Chlamydia, Glandular Fever, Recurrent abscess, Current abcess to both arms, Abscess under arm removal | Hepatitis B positive, Mumps (caused deafness) |  |
| Immunological Conditions | HIV Positive, Lupus Antibodies |  |  | Multiple Allergies, Antiphospholipid syndrome, Cold Urticaria | Psoariesis Arthritis |  |
| Dermatological Conditions | Eczema, Psoriasis |  |  | Psoriasis, Dermatitis, Ecezma |  |  |
| Congenital Conditions | Congenital Clicky Hip, Congenital Absent Kidney, Double Uterus and Cervix, Talipes |  |  |  |  |  |
| Trauma-Related Conditions | Trauma to Left Arm, Lost Right Leg |  |  | Tonsillectomy, Grommets, Dilation and Curettage (D&C), Microsurgery |  |  |
| Cancer-Related Conditions | Pneumonectomy for Carcinoid Tumour, Cervical Neoplasm |  | Thyroidectomy for Malignancy | Melanoma, Osteosarcoma, Non-Hodgkin's Lymphoma (NHL) | Melanoma |  |
| Others | Chronic Fatigue Syndrome, No-specific itching, Past Alcohol and Amphetamine Abuse, IV Drug Abuser |  | Chronic Fatigue Syndrome | Chronic Fatigue Syndrome, Wisdom Teeth Removal, Reflux - due to pregnancy, Tonsillectomy, Tonsills and Adeniuds Removal, Breech Presentation with Current Pregnancy, Grommets, Breast Reduction, Eye Operation, Tumour under left finger | Infundibulation and repair of same |  |

*Table S7: Complete-case IPD Meta-analysis results (log ORs, ORs, p-values, confidence intervals on OR scale, and the results of heterogeneity check) of risk factors on the Adverse Pregnancy Outcome (Multivariable Analysis).*

| Risk Factor | Log-OR | Adjusted OR | P-Value | CI (OR scale) | Tau^2^ | I^2^ (%) | Q-statistic P-value | Egger test p-value |
| --- | --- | --- | --- | --- | --- | --- | --- | --- |
| Past medical history (PMH)^a^ | 0.84 | 2.31 | 0.024 | (1.12, 4.78) | 0.3566 | 46.54 | 0.074 | 0.594 |
| Cigarette smoking^b^ | 0.86 | 2.37 | 0.033 | (1.07, 5.23) | 0.2171 | 26.86 | 0.267 | 0.754 |
| Abnormal Fetal Heart Rate^b^ | 1.58 | 4.86 | <0.001 | (2.18, 10.80) | 0.0000 | 0.00 | 0.158 | 0.097 |
| Duration of RFM^c^ | 0.003 | 1.003 | 0.159 | (0.999, 1.006) | 0.0000 | 0.00 | 0.588 | 0.847 |
| EFW percentile^d^ | -0.03 | 0.97 | 0.0281 | (0.95, 0.99) | 0.0007 | 83.99 | <0.001 | 0.771 |
| ^a^Adjusted for maternal age.  ^b^Adjusted for maternal age, ethnicity, and PMH.  ^c^Adjusted for maternal age, bmi, and EFW centile.  ^d^Adjusted for maternal age, ethnicity, PMH, gestation at RFM presentation | | | | | | | | |

*Table S8: Results of sensitivity analyses by excluding the intervention arm from RCTs (adjusted ORs, 95% confidence intervals on OR scale, p-values, and the Q-statistic p-value of heterogeneity check) of risk factors on the Adverse Pregnancy Outcome (based on different sensitivity analyses and data used).*

|  | Main IPD-MA | | | | Sensitivity Analysis  (excluding the intervention arms) | | | |
| --- | --- | --- | --- | --- | --- | --- | --- | --- |
| Risk Factor | Adjusted OR | 95% CI (OR scale) | P-Value | Q-statistic P-value | Adjusted OR | 95% CI (OR scale) | P-Value | Q-statistic P-value |
| Past Medical History (PMH)^a^ | 2.35 | (1.14, 4.82) | **0.020** | 0.076 | 2.27 | (1.10, 4.69) | **0.026** | 0.091 |
| Cigarette Smoking^b^ | 2.96 | (1.36, 6.44) | **0.006** | 0.174 | 2.20 | (0.95, 5.10) | **0.065** | 0.219 |
| Abnormal Fetal Heart Rate^b^ | 3.65 | (1.84, 7.23) | **<0.001** | 0.311 | 4.19 | (2.22, 7.92) | **<0.001** | 0.565 |
| Duration of RFM^c^ | 1.003 | (0.999,1.006) | 0.129 | 0.633 | 1.003 | (0.999, 1.006) | 0.135 | 0.485 |
| EFW Percentile at RFM Presentation^d^ | 0.97 | (0.95, 0.99) | **0.005** | **<0.001** | 0.97 | (0.95, 0.99) | **<0.001** | **0.005** |
| ^a^Adjusted for maternal age.  ^b^Adjusted for maternal age, ethnicity, and PMH.  ^c^Adjusted for maternal age, bmi, and EFW centile.  ^d^Adjusted for maternal age, ethnicity, PMH, gestation at RFM presentation | | | | | | | | |

**Figures**


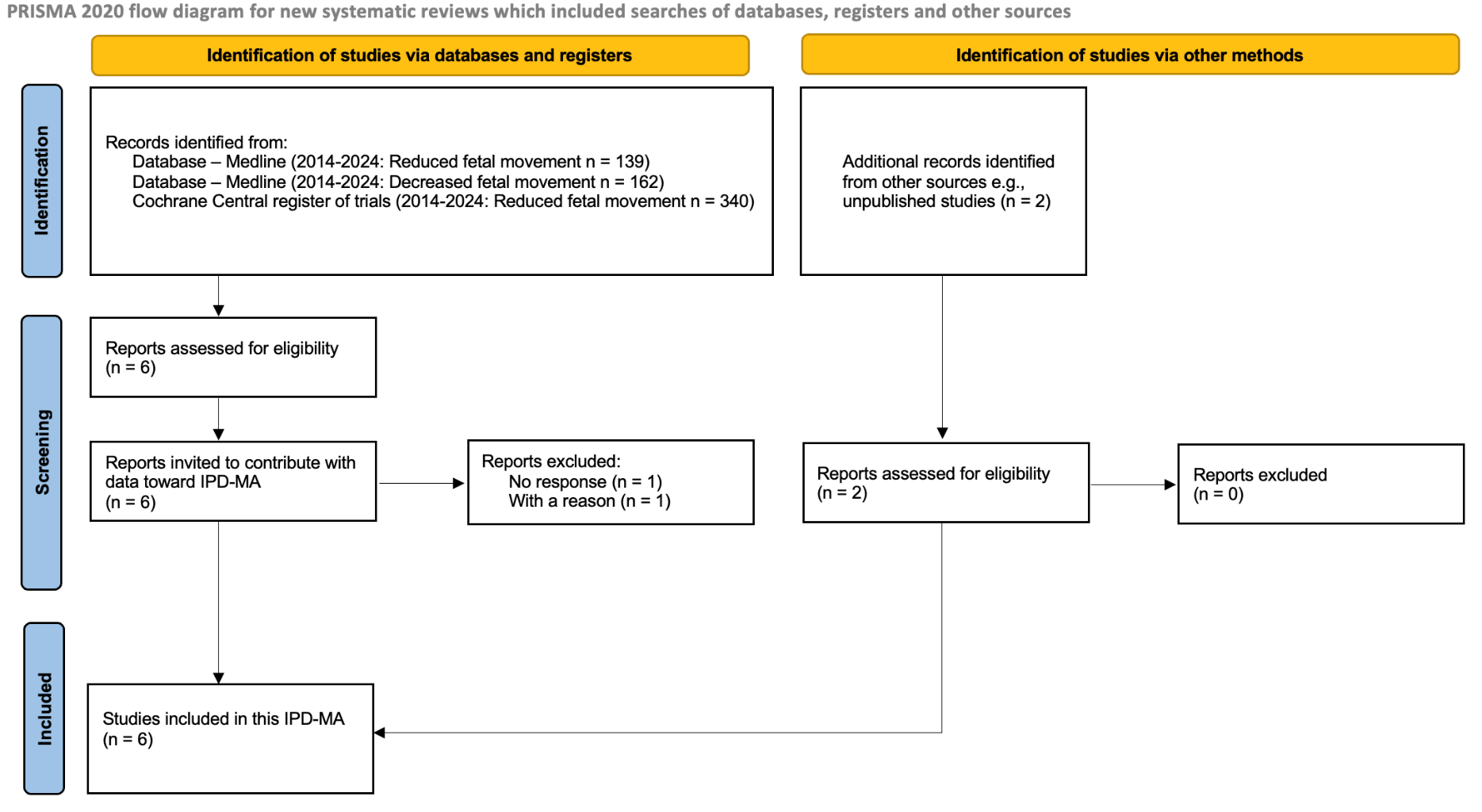


*Figure S1: PRISMA flow diagram for study identification.*


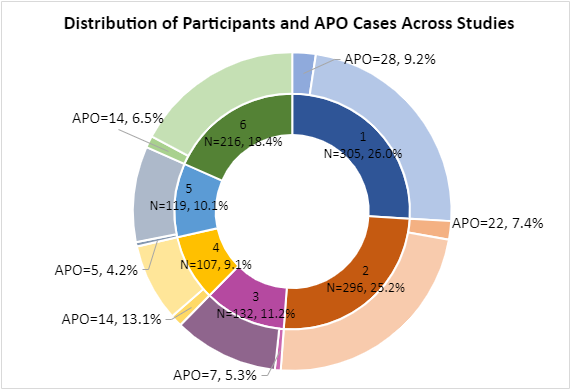


*Figure S2: Sunburst chart showing the distribution of participants and cases of APO across six studies. The inner ring of the chart shows the total number of participants in each study, while the outer ring provides the corresponding number and percentage of APO cases for each study.*
